# Loss of Nocturnal Dipping in Type 2 Diabetes Mellitus According to Diabetic Kidney Disease: The Role of Vitamin D Levels

**DOI:** 10.3390/nu18172807

**Published:** 2026-08-27

**Authors:** João Soares Felício, Isabel Jacob Fernandes, Marina Izabel Monteiro de Oliveira, Beatriz Dias Lobo, Maria Eduarda Nunes Avelar do Carmo, Alessandra Pitanga Cardoso Costa, Isabella Almeida Santos, Karem Mileo Felício, Priscila Boaventura Barbosa De Figueiredo, Lilian de Souza D’Albuquerque Silva, Franciane Trindade Cunha de Melo, Ana Carolina Contente Braga de Souza, Márcia Costa dos Santos, Lorena Vilhena De Moraes, Mayana Batista Barros, Paula Gabriella Costa da Penha, Ana Clara Fleury da Fonseca Rodrigues, Fábio Feijó, Pedro Paulo Piani, Valéria Suênya Leal, Melissa de Sá Oliveira dos Reis, Ana Regina Bastos Motta, Natércia Neves Marques De Queiroz

**Affiliations:** Endocrinology Department, University Hospital João de Barros Barreto, Federal University of Pará, Belem 66075-110, Brazil; bebeljfernandes3@gmail.com (I.J.F.); izabel.marina@hotmail.com (M.I.M.d.O.); beatrizxlob@gmail.com (B.D.L.); eduardaavelar05@gmail.com (M.E.N.A.d.C.); lelepitangacc@gmail.com (A.P.C.C.); bella.santos2001@terra.com.br (I.A.S.); karemfelicio@yahoo.com.br (K.M.F.); pribfigueiredo@gmail.com (P.B.B.D.F.); liliandalbuquerque@yahoo.com.br (L.d.S.D.S.); franciane.melo@hotmail.com (F.T.C.d.M.); carol.souza25@hotmail.com (A.C.C.B.d.S.); marciacsantos29@gmail.com (M.C.d.S.); lorena.v.moraes@gmail.com (L.V.D.M.); dramayanabarros@gmail.com (M.B.B.); paula.penha@hotmail.com (P.G.C.d.P.); anafleuryy@gmail.com (A.C.F.d.F.R.); fabiofeijo05@hotmail.com (F.F.); pedropiani@yahoo.com.br (P.P.P.); valeriasuenya@gmail.com (V.S.L.); melissabiomedica1@gmail.com (M.d.S.O.d.R.); arbmotta@gmail.com (A.R.B.M.); natercianeves@hotmail.com (N.N.M.D.Q.)

**Keywords:** nocturnal dipping, vitamin D, type 2 diabetes mellitus, diabetes kidney disease, cardiovascular autonomic neuropathy

## Abstract

**Background/Objectives:** Abnormal nocturnal dipping (ND) in diabetes mellitus is linked to adverse outcomes. Its association with vitamin D (VD), cardiovascular autonomic neuropathy (CAN), and diabetic kidney disease (DKD) remains unclear. We aim to evaluate the association of VD status with ND loss as well as CAN in type 2 diabetes mellitus (T2DM) across different DKD stages. **Methods:** 83 T2DM patients were included in this cross-sectional study: 36 with early-stage DKD (urine albumin-to-creatinine ratio [UACR] 30–299 mg/g; Group 1) as well as 47 with advanced-stage DKD (UACR ≥ 300 mg/g; Group 2). **Results:** Patients with advanced DKD exhibited higher CAN prevalence (72.70% vs. 52.80%; *p* < 0.05), lower 25(OH)D (26.90 ± 9.90 vs. 29.60 ± 8.00 ng/mL; *p* = 0.05), and lower systolic ND (0.82 ± 8.40 vs. 4.50 ± 7%; *p* < 0.05). Among patients with advanced DKD, lower 25(OH)D levels, classified according to the Institute of Medicine (IOM) criteria (normal ≥ 20 ng/mL), were associated with reduced systolic ND (1.90 ± 7.80 vs. −5.60 ± 9.30%; *p* < 0.05), while the Endocrine Society criteria showed a progressive reduction in systolic ND across VD classification (insufficiency 20–29.9, deficiency <20, and sufficiency ≥30 ng/mL), (−5.60 ± 9.30 vs. −0.20 ± 7.30 vs. 3.30 ± 8%, respectively; *p* < 0.05). Patients with definite/severe CAN exhibited lower systolic ND (−4.38 ± 7.41 vs. 3.34 ± 8.00%; *p* < 0.01) than those with absent/early CAN. In addition, lower VD levels were associated with greater CAN severity according to both the Endocrine Society criteria (*p* < 0.01, r = 0.40), and the IOM criteria (*p* < 0.05, r = 0.30). In the regression model, VD remained an independent predictor of absence of diastolic ND (Rsqr = 0.393; *p* < 0.05). **Conclusions:** These findings suggest a potential link between vitamin D status, autonomic dysfunction, and ND in patients with T2DM and advanced DKD.

## 1. Introduction

Nocturnal dipping (ND) corresponds to the physiological reduction in blood pressure (BP) during sleep and represents one of the main parameters used to assess circadian BP behavior [1,2,3,4]. A nighttime drop in blood pressure of at least 10% defines the dipper pattern according to ambulatory blood pressure monitoring (ABPM) [2,3]. Abnormal ND, defined as a ND reduction of less than 10%, has been related with the development of kidney disease and also adverse cardiovascular outcomes in individuals affected by diabetes [5,6,7,8,9,10,11].

Among the factors potentially involved in impaired ND, diabetic kidney disease (DKD) and cardiovascular autonomic neuropathy (CAN) have received particular attention. DKD, characterized by progressive renal dysfunction, albuminuria, and impaired vitamin D activation [12,13,14], has been increasingly recognized as an important contributor to disruption of the physiological circadian BP pattern, including reduced ND and a higher frequency of nocturnal hypertension in people who have diabetes [6,8,15,16,17,18,19,20]. Conversely, impaired ND has also been found to be a reliable predictor of DKD onset and progression, suggesting a bidirectional relationship between renal dysfunction and circadian BP abnormalities [18]. In parallel, CAN, characterized by the Toronto Consensus as impairment of cardiovascular autonomic control in diabetes, is associated with sympathetic overactivity and reduced heart rate variability [11,21], with a well-established association with impaired ND, reverse-dipping and non-dipping patterns in individuals affected by diabetes [22,23,24,25,26,27]. CAN and DKD appear to be closely interconnected complications of diabetes [21,25,28,29,30,31].

Furthermore, vitamin D deficiency has been identified as a possible cause of both renal and autonomic dysfunction in diabetes, with lower 25-hydroxyvitamin D (25(OH)D) levels associated with DKD progression, higher levels of albuminuria as well as a higher prevalence of CAN [27,31,32,33,34,35,36,37,38,39,40,41,42,43,44,45]. Despite its traditional function in mineral metabolism, vitamin D has been linked to the control of the renin–angiotensin–aldosterone pathway, inflammatory pathways, endothelial function, and autonomic cardiovascular control [9,13,14,46,47,48,49]. Additionally, emerging evidence suggests that VD status may also influence circadian BP regulation in non-diabetic populations [50]. In Type 1 DM (T1DM), we have recently reported a connection between lower serum vitamin D levels, the existence of CAN, and absence of ND, as well as improvements in autonomic parameters following vitamin D supplementation [27].

Despite evidence linking vitamin D deficiency to DKD, CAN, and impaired ND regulation, the interplay among these factors across the spectrum of DKD remains poorly understood. Based on the available literature, no previous study has simultaneously evaluated vitamin D levels, CAN severity, as well as ND in individuals with Type 2 DM (T2DM) according to the DKD stage.

## 2. Materials and Methods

### 2.1. Study Design and Patients

A cross-sectional study was conducted to investigate the relationship among serum VD levels, CAN, as well as ABPM findings, including nocturnal dipping status and blood pressure variability, in individuals with T2DM at both early and advanced phases of DKD. A total of 83 participants receiving regular care at the Endocrinology Department of the João de Barros Barreto University Hospital (HUJBB), Federal University of Pará (UFPA), were enrolled between 2023 and 2025. Participants were categorized based on urinary albumin-to-creatinine ratio (UACR) values in an early DKD group (30–299 mg/g; *n* = 36), corresponding to KDIGO 2022 stage A2, and an advanced DKD group (≥300 mg/g; *n* = 47), corresponding to stage A3. The study protocol complied with the ethical principles established by the Declaration of Helsinki (2008 revision) as well as the Nuremberg Code and received approval from the HUJBB Research Ethics Committee (number of reference 88974918.6.0000.0017). All participants signed an Informed Consent Form (ICF). Eligibility criteria included: (a) signing the ICF before the study participation; (b) T2DM diagnosis and age ≥30 with routine endocrinologist follow-up; (c) stable therapy using glucose-lowering oral agents and/or using therapy with insulin for a minimum of three months; (d) presence of DKD with UACR ≥ 30 mg/g; (e) 25 to 90 mL/min/1.73 m^2^ as the estimated glomerular filtration rate (GFR); (f) steady dosages of every drug for a minimum of four weeks before the screening appointment; and (g) capacity and willingness to comply with all study procedures, attend scheduled visits, and complete the study.

Exclusion criteria were: (a) type 1 diabetes mellitus or other types of diabetes; (b) bone metabolism diseases; (c) previous hepatic disease; (d) taking vitamin D as well as calcium supplementation in the past three months prior to screening; (e) uncompensated hypothyroidism or hyperthyroidism; (f) lactation, pregnancy, or the intention to become pregnant; (g) diseases that, at the researcher’s discretion, could interfere with life expectancy; and (h) abuse of recreational drugs or alcohol that may interfere with study procedures or participant safety.

### 2.2. Data Collection

Following eligibility assessment, participants underwent clinical, laboratory, and blood pressure evaluations during scheduled visits at the Clinical Research Department of HUJBB. Demographic data, medical history, use of medication, as well as anthropometric metrics (mass, stature, and body mass index) were collected. The diagnosis of T2DM followed established clinical and laboratory criteria [51].

Serum 25(OH)D levels were assessed by the DiaSorin LIAISON^®^ chemiluminescence method (DiaSorin, Stillwater, MN, USA) [52], a technique validated via the Vitamin D External Quality Assessment Scheme (DEQAS), a widely used international external quality assessment program for vitamin D testing. Glycated hemoglobin (HbA1c) was determined by efficient liquid chromatography (HPLC) [53]. Fasting plasma glucose, albumin, lipid profile, and triglycerides were analyzed by automated colorimetric techniques. Serum creatinine was determined by an automated kinetic method, and albuminuria was measured by immunoturbidimetry [54]. The GFR was calculated with the CKD-EPI formula, without race adjustment [55].

### 2.3. Classification of DKD

UACR was assessed three times in all patients, with each measurement occurring at different time intervals. For DKD classification, two categories were considered: moderately increased (UACR 30–299 mg/g) and significantly increased albuminuria (UACR ≥ 300 mg/g) [12]. Patients were categorized based on the classification identified in at least two of the three measurements. For consistency and clarity during the manuscript, participants exhibiting moderately elevated and significantly elevated albuminuria were categorized as having early and advanced phases of diabetic kidney disease, respectively.

### 2.4. Vitamin D Assessment

Serum 25(OH)D concentrations were categorized based on two reference guidelines: the Institute of Medicine (IOM) [56], which categorizes 25-hydroxyvitamin D [25(OH)D] levels into deficiency (<20 ng/mL) and sufficiency (≥20 ng/mL), and the historical 2011 Endocrine Society criteria [57], that stratify 25(OH)D serum levels into insufficiency (20–29.90 ng/mL), deficiency (<20 ng/mL), and sufficiency (≥30 ng/mL).

### 2.5. Ambulatory Blood Pressure Monitoring (ABPM)

The oscillometric method was used for ambulatory blood pressure monitoring (ABPM) with a validated portable monitor (Oscar 2 ABPM SphygmoCor^®^), with measurements taken every 15 min over a 24 h period. Instructions were given to the participants to keep their routine and to register their sleep as well as wake periods. Mean systolic and diastolic BP (SBP and DBP) values were obtained for the 24 h, during the waking and sleeping periods. ABPM has previously demonstrated good reproducibility in patients with T2DM [58,59]. The examination was considered valid when ≥70% of the scheduled measurements were successfully obtained. Morning blood pressure was defined as the average of the measurements obtained during the first two hours after awakening [60].

Nocturnal dipping (ND) was determined through the percentage reduction in blood pressure during sleep relative to the daytime period, using the following well-established and standard formula Systolic or diastolic nocturnal dipping: (%) = [(mean waking SBP/DBP − mean sleeping SBP/DPB)/mean waking SBP] × 100 [59,61]. Participants were classified as reverse dipper (<0%), non-dipper (0–10%), dipper (10–20%), and extreme dipper (>20%) [8]. For additional analyses, individuals were also grouped in two more patterns: loss of ND (ND < 0%) and presence of ND (ND > 0%).

### 2.6. CAN Assessment

Autonomic functional testing was conducted in the morning following verification of capillary glucose concentrations between 70 and 250 mg/dL. Participants were instructed to avoid alcoholic beverages, caffeine, as well as smoking for at least 8 h and vigorous physical activity for 24 h prior to testing. Cases of fever (>37.80 °C), significant emotional stress, or recent hypoglycemia resulted in rescheduling of the examination. The VNS-Micro^®^ system (Neurosoft LLC, Ivanovo, Russia) was used.

Cardiovascular autonomic reflex tests (CARTs) served as the reference standard in line with the Toronto Consensus, which included deep breathing (E:I ratio), the Valsalva maneuver, and the orthostatic test (30:15 ratio and assessment of orthostatic hypotension) [21]. For the Valsalva maneuver, the patient sustained an expiratory strain of 40 mmHg for 15 s while lying supine under continuous electrocardiographic tracking. The 30:15 test evaluated the relationship between early heart rate elevation and subsequent reflexive deceleration following the change to the standing position. The deep breathing test consisted of determining the E:I ratio during controlled respiratory cycles. Diagnostic criteria for orthostatic hypotension required a fall of ≥20 mmHg in systolic blood pressure and/or ≥10 mmHg in diastolic blood pressure within a 3 min standing interval. Time-domain indices of heart rate variability (HRV) were gathered in supine patients through the VNS-Micro^®^ platform (Neurosoft LLC, Ivanovo, Russia) [62]. For spectral analysis, participants remained elevated in the supine position at 30° and breathing spontaneously during a 300 s ECG recording. Signals were digitally transformed using mathematical algorithms, generating amplitude-versus-frequency spectra.

The following spectral bands were evaluated: very low frequency (VLF: 0.01–0.04 Hz), representing vasomotor sympathetic activity and body temperature regulation; low frequency (LF: 0.04–0.15 Hz), associated with sympathetic–vagal baroreflex modulation; as well as high frequency (HF: 0.15–0.5 Hz), considered a marker of parasympathetic activity. In the time domain, RRmin, RRmax, RRNN, and SDNN were analyzed. Frequency-domain parameters included VLF, LF, HF, total power (TP), alongside the LF/HF ratio, used as an indicator of sympathovagal balance.

Interpretation of HRV parameters followed the reference values proposed by [62], which should be considered estimates without validated clinical cutoff points. Identification of CAN followed the framework outlined by the Toronto Consensus criteria. For CAN staging, classification was based on the number of abnormal Ewing tests and the presence or absence of orthostatic hypotension. Patients without evidence of CAN were defined by normal test results. Early CAN was defined by one abnormal heart rate test (or borderline results), whereas definite CAN was diagnosed upon the detection of two or more abnormal tests. Severe CAN was diagnosed in the presence of orthostatic hypotension associated with at least one abnormal autonomic test [21].

### 2.7. Statistical Analysis

Computational processing was conducted using SigmaPlot 12.0^®^ (Systat Software Inc., San Jose, CA, USA) alongside Statistical Package for the Social Sciences (SPSS version 22, IBM Corp., Chicago, IL, USA). A *p* value < 0.05 was defined as statistically significant throughout all evaluations.

Qualitative data were summarized as absolute frequencies accompanied by percentages (%). Quantitative data are presented as mean ± standard deviation when normally distributed and as median and interquartile range (25th–75th percentile) when non-normally distributed. The distribution of continuous variables was assessed using the Shapiro–Wilk test.

For comparative analyses of continuous variables between the early-stage and advanced-stage DKD groups, Student’s independent-samples *t*-test was used for normally distributed variables, whereas the Mann–Whitney U test was used for variables with a non-normal distribution. The same approach was applied to other analyses involving two independent groups, including comparisons of ABPM parameters according to the Institute of Medicine vitamin D classification (25[OH]D < 20 vs. ≥20 ng/mL), comparisons between patients with absent/early versus definite/severe cardiovascular autonomic neuropathy (CAN), and comparisons between reverse dippers and participants with other nocturnal dipping patterns.

For analyses involving three independent groups, specifically comparisons of ABPM parameters across the historical 2011 Endocrine Society vitamin D categories (sufficiency, ≥30 ng/mL; insufficiency, 20–29.9 ng/mL; and deficiency, <20 ng/mL), one-way ANOVA was used when the assumptions of normality were satisfied, whereas the Kruskal–Wallis test was used for non-normally distributed variables.

Associations between categorical variables, including the comparison of clinical characteristics, comorbidities, medication use, CAN prevalence, and nocturnal dipping categories between DKD groups, were evaluated using the chi-square test. Fisher’s exact test was used instead when the assumptions of the chi-square test were not met because of small expected cell frequencies.

Multivariable logistic regression analysis was performed to investigate whether serum 25(OH)D concentration was independently associated with reverse-dipping status. Reverse dipping was entered as the dependent binary variable (reverse dipper = 1; non-dipper, dipper, and extreme dipper = 0), and serum 25(OH)D concentration was entered as the main independent variable. The multivariable model was adjusted for potential confounding factors, including age, duration of T2DM, HbA1c, eGFR, lipid profile, blood pressure levels, and the use of ACE inhibitors/angiotensin II receptor blockers, SGLT2 inhibitors, beta-blockers, and GLP-1 receptor agonists.

Sample power and size were calculated according to the primary study hypothesis evaluating the relationship between serum 25-hydroxyvitamin D levels and nocturnal blood pressure dipping. Considering an expected Spearman correlation coefficient of 0.30, a two-sided significance level of 5% (α = 0.05), and a statistical power of 80% (1 − β), the minimum required sample size was estimated to be 70 participants. Sample size calculations were performed using SigmaPlot version 12.0.

## 3. Results

A total of 83 individuals took part in the study, with their main clinical and demographic data detailed in Table 1. Participants were divided into an early DKD stage (30–299 mg/g; *n* = 36) and an advanced DKD stage (≥300 mg/g; *n* = 47). The comparisons were performed between these groups. As expected, patients with advanced DKD were older and exhibited a longer history of T2DM. Notably, all participants in the advanced DKD group were receiving SGLT2 inhibitors. In addition, they exhibited a greater burden of diabetes-related complications, including a higher CAN prevalence.

Table 2 shows a laboratory profile consistent with the greater severity of renal dysfunction observed in patients with advanced DKD, including higher serum creatinine and UACR levels and lower GFR values. In addition, these patients showed significantly decreased circulating 25(OH)D levels compared to subjects with early-stage DKD, in conjunction with a less favorable lipid profile, characterized by elevated total cholesterol.

BP parameters obtained from 24 h ABPM are presented in Table 3. Patients with advanced-stage DKD exhibited higher SBP levels throughout the 24 h period, including daytime, nighttime, and morning intervals, along with reduced nocturnal systolic BP dipping. Importantly, mean nocturnal systolic BP dipping in this group fell within a non-dipper range.

The blood pressure parameters presented above were further grouped according to 25(OH)D levels according to guidelines from the Institute of Medicine (IOM) as well as the Endocrine Society (Table 4 and Table 5). Similar results were obtained using both classification systems. Among participants with advanced-stage DKD, lower circulating VD levels were associated with reduced nocturnal SBP dipping, and a trend toward a lower nocturnal DBP dipping, whereas no such correlation was found in patients with early-stage DKD. No significant differences were found for the remaining BP variables across VD categories.

At the advanced-DKD stage, patients were categorized as reverse dippers (*n* = 16; 34%), non-dippers (*n* = 18; 38.30%), dippers (*n* = 11; 23.40%), or extreme dippers (*n* = 2; 4.30%) according to ND status. In this group, compared with the other dipping patterns, reverse dippers showed lower serum 25(OH)D concentrations (26.70 ± 9.30 vs. 30.90 ± 7.60, *p* < 0.05) and higher triglyceride levels (303 ± 205 vs. 190.40 ± 104, *p* < 0.05).

In the early-stage DKD group, nine patients (25%) were classified as reverse dippers, 18 (50%) as non-dippers, nine (25%) as dippers, and none as extreme dippers. The distribution of nocturnal dipping patterns did not differ significantly between the early- and advanced-stage DKD groups (*p* = 0.43).

BP parameters according to CAN severity, classified based on the Toronto Consensus criteria, are presented in Table 6. Among patients with advanced-stage DKD, those with definite/severe CAN (*n* = 19; 40.43%) exhibited significantly lower systolic and diastolic ND values, compared with patients with absent/early CAN (*n* = 28; 59.57%). Notably, patients with definite/severe CAN exhibited a systolic reverse-dipper pattern. The remaining ambulatory monitoring parameters did not differ significantly between study groups.

To investigate these relationships more thoroughly, associations were assessed using Spearman correlation. VD concentration was positively correlated with diastolic BP dipping (ρ = 0.30; *p*-value < 0.05). Additionally, a multivariable logistic regression model was fitted with ND status as the outcome variable (reverse dippers = 1; non-dippers, dippers, and extreme dippers = 0), and VD concentration as the predictor variable. VD concentration retained its independent association with absence of diastolic ND, after adjustment for T2DM duration, age, lipid profile, HbA1c, eGFR, blood pressure levels, ACEi/ARB use, SGLT2i, beta-blockers and GLP-1 receptor agonist (β = −0.0266; Rsqr = 0.393, *p* < 0.05). These results were not observed in patients with early-stage DKD.

To further investigate the association of vitamin D status with CAN, Spearman correlation analyses were performed according to the Toronto Consensus classification (0 = absent, 1 = early, 2 = definite, and 3 = severe CAN). A significant positive correlation was observed between worsening vitamin D categories defined using the Endocrine Society criteria (sufficiency, insufficiency, and deficiency) and increasing CAN severity (r = 0.412, *p* < 0.05). A comparable pattern was observed when VD status was categorized according to Institute of Medicine criteria (normal vs. deficient), although with a lower correlation coefficient (r = 0.317, *p* < 0.05). These results suggest a graded relationship between worsening VD status and greater CAN severity, regardless of the vitamin D classification system used.

## 4. Discussion

Our results show an association between lower serum vitamin D (VD) concentrations, presence and intensity of cardiovascular autonomic neuropathy (CAN), and impaired nocturnal dipping (ND) in patients with type 2 diabetes mellitus (T2DM) and advanced diabetic kidney disease (DKD). Only in this group, a progressive decline in nocturnal systolic blood pressure (SBP) dipping was observed as vitamin D status worsened, regardless of whether vitamin D status was classified according to Institute of Medicine or Endocrine Society criteria. Furthermore, individuals with definite/severe CAN exhibited lower systolic and diastolic ND values compared with those with absent/early CAN. Lower vitamin D concentrations were also associated with both greater CAN severity and reduced nocturnal diastolic blood pressure (BP) dipping, as demonstrated by correlation analyses. Notably, serum vitamin D concentration remained an independent predictor of diastolic reverse dipping after adjustment for potential confounding factors.

Abnormal ND patterns have frequently been correlated with a higher risk of cardiovascular and renal events and have been recognized as a marker of adverse prognosis [17,63,64,65,66]. Similar associations have been reported in populations with T2DM and DKD [7,10,67,68]. Recently, Chiriacò et al. (2022), in a retrospective cohort study involving 349 patients with diabetes mellitus, demonstrated that reverse-dipper pattern was associated with reduced survival and a twofold increase in all-cause mortality [67]. Likewise, Akintunde et al. (2024), in a cross-sectional study with 100 T2DM patients, reported associations between non-dipping and reverse-dipping patterns and markers of increased cardiovascular risk, including impaired kidney function [68]. In light of these findings, ABPM remains the reference method for assessing 24 h BP behavior, enabling the detection of abnormalities in ND and sleep-time BP that are not identified by office measurements and that provide important prognostic information. Accordingly, characterization of ND patterns may improve renal and cardiovascular risk assessment in patients with diabetes.

As expected, patients with early-stage DKD exhibited a lower frequency of diabetes-related complications and less severe renal impairment compared with those with advanced DKD. Although participants with early-stage DKD also presented a high prevalence of the non-dipper pattern, consistent with previous studies reporting alterations in nocturnal dipping behavior in this population [69,70], the association among reduced ND, lower vitamin D concentrations, and the presence of CAN was not significant in the setting of early renal disease. These results might reflect the lower degree of renal and autonomic impairment in these individuals, as abnormalities in blood pressure regulation and cardiovascular autonomic function tend to become more pronounced with DKD progression [2,12,21,71].

Abnormal nocturnal dipping patterns have consistently been reported in individuals with diabetes and greater renal impairment, characterized by increased albuminuria and a less favorable BP profile compared with the dipper pattern [6,15,16,17,69,72]. More recent evidence has further linked progression of renal injury to increased BP variability [20]. In a prospective study of individuals with T1DM and initially normal albumin excretion, Lurbe et al. demonstrated that elevated sleep-time systolic BP preceded the development of microalbuminuria [6]. In parallel, Felício et al. (2010) also described similar results in T2DM [17]. Finally, studies in T2DM have reported a higher prevalence of albuminuria among patients with a systolic non-dipper pattern [16,69,72]. From a pathophysiological perspective, persistent sympathetic activation during sleep, reflected by increased norepinephrine levels, may contribute to the maintenance of vascular resistance and elevated BP in patients with diabetic nephropathy [15]. In addition, sodium dysregulation, fluid retention and persistent activation of the renin–angiotensin–aldosterone system (RAAS), commonly observed in DKD, may promote volume overload and sustained nocturnal hypertension [73]. Consistent with these observations, patients with advanced DKD in our study exhibited higher systolic BP levels throughout the 24 h period, along with reduced nocturnal systolic BP dipping compatible with a non-dipper profile. Collectively, these findings support the association between adverse ND behavior and greater severity of renal involvement in diabetes mellitus.

Beyond the hemodynamic and renal mechanisms discussed above, cross-sectional studies have demonstrated associations between CAN, autonomic dysfunction, and non-dipper and reverse-dipper blood pressure patterns in individuals with diabetes [22,23,24,26,27]. In different populations of patients with diabetes, including both T1DM and T2DM, Spallone et al. demonstrated that CAN is a major determinant of attenuated nocturnal blood pressure decline, whereas the absence of nocturnal dipping, particularly of systolic blood pressure, exhibits high specificity for identifying this complication [22,23,24,27]. The present data reinforce the strong association between cardiovascular autonomic dysfunction and disturbances in circadian regulation of blood pressure in individuals with diabetes mellitus. Additionally, Kim et al. (2019), in a study including 45 patients with T2DM and 16 healthy controls, observed that progressive reduction in nocturnal blood pressure decline described in patients with diabetes and microvascular complications was accompanied by impaired baroreflex sensitivity, further supporting the role of autonomic dysfunction in abnormalities of nocturnal blood pressure regulation [26]. Several pathophysiological mechanisms may underlie the association between abnormal ND and CAN [21,25,26,74,75]. CAN reflects structural and functional impairment of sympathetic and parasympathetic fibers, resulting in autonomic imbalance characterized by reduced vagal modulation and relative sympathetic predominance during sleep, which may contribute to altered nocturnal cardiovascular regulation [26,74,75]. Under physiological conditions, sleep is accompanied by reduced sympathetic tone and predominance of parasympathetic activity, favoring decreases in heart rate, peripheral vascular resistance, and blood pressure. In the presence of CAN, however, this autonomic modulation is impaired, promoting persistent nocturnal sympathetic activity and attenuation of the physiological ND decline [21,25]. Consistent with these mechanisms, greater CAN severity in the present study was associated with abnormal ND among patients with advanced DKD, with individuals classified as having definite/severe CAN exhibiting lower dipping values than those with absent/early CAN.

Furthermore, both greater CAN severity and reduced nocturnal DBP dipping were associated with lower VD levels, implying a potential interaction between VD deficiency and cardiovascular autonomic dysfunction in diabetes mellitus. Reduced 25(OH)D concentrations were independently associated with greater CAN severity and reduced ND, particularly among individuals with advanced DKD. These results are consistent with substantial evidence linking vitamin D deficiency to both autonomic impairment [27,32,33,34,35,36] and diabetic kidney disease progression [37,38,39,40,41,42,43,44,45]. Among these findings, our group has recently shown associations between lower vitamin D concentrations and the presence of CAN in 76 individuals with type 2 diabetes mellitus and advanced DKD [31], as well as improvements in autonomic parameters and reductions in awake SBP and morning SBP surge after vitamin D supplementation in T1DM patients [27]. Although our results are in agreement with these previous observations, the available evidence remains limited and heterogeneous, with findings varying across different clinical populations [76,77,78,79,80,81]. In a recent retrospective cohort including 585 hypertensive individuals stratified according to nocturnal blood pressure decline patterns, non-dippers exhibited calcium-phosphate dysregulation, characterized by increased parathyroid hormone levels and reduced VD concentrations compared with those classified as dippers [50]. Despite the fact that this study was conducted in a non-diabetic population without DKD or CAN, the authors suggested the potential value of investigating biomarkers such as vitamin D in the assessment of circadian blood pressure regulation.

Although the mechanisms involved in the relationship between VD and ND remain unclear, the integration of available evidence supports diverse pathophysiological hypotheses. Vitamin D levels have been correlated with blood pressure regulation in different clinical settings, including diabetic kidney disease, suggesting that VD may influence cardiovascular and renal pathways involved in BP control [82,83]. Several of the mechanisms known to determine the nocturnal dipping pattern are also influenced by vitamin D metabolism and signaling. Among these, autonomic modulation and the RAA system play crucial roles in nocturnal dipping [1,15,21,84]. Disturbances in these regulatory systems have consistently been associated with impaired nocturnal dipping decline [15,85,86]. Interestingly, studies demonstrated that VD levels could contribute to the regulation of autonomic and RAAS activity, with experimental and clinical evidence suggesting that VD deficiency may be associated with sympathetic overactivity, autonomic imbalance, and increased RAAS activation, partly through reduced suppression of renin expression [13,48,87,88,89]. Specifically, in patients with diabetes, some cross-sectional studies also show these connections [90,91].

Beyond neurohormonal regulation, endothelial dysfunction, oxidative stress, and chronic low-grade inflammation are well-established factors that contribute to abnormal nocturnal blood pressure behavior. Impaired endothelial-dependent vasodilation, increased production of reactive oxygen species, and persistent inflammatory activation can compromise vascular adaptation to the sleep period, thereby attenuating the physiological nocturnal decline in blood pressure [1,4,21,92,93,94]. Notably, these same processes have also been linked to vitamin D deficiency, with pleiotropic effects on vascular homeostasis, including the maintenance of endothelial function, attenuation of oxidative stress, and modulation of inflammatory responses. Reduced vitamin D availability could lead to endothelial impairment, increased oxidative burden, and enhanced inflammatory activity, creating a biological milieu that may favor the development of an impaired nocturnal blood pressure decline [46,84,95,96,97,98,99]. In addition, alterations in these same pathways have been described in both CAN [21,25,46,100,101,102] and DKD [13,48,96], suggesting the existence of shared mechanisms linking vitamin D deficiency, autonomic dysfunction, impaired nocturnal BP decline, and development of renal damage in individuals with diabetes.

The primary limitation of the present study is the relatively limited sample size, and larger studies are necessary to confirm the results. Additionally, due to its cross-sectional design, causal relationships among serum vitamin D concentrations, CAN, and ND cannot be established, and reverse causality cannot be excluded. All T2DM patients were followed in the Endocrinology Department of our University Hospital and had their physical activity and dietary intake previously standardized according to the American Diabetes Association guidelines [51], thereby minimizing the influence of those factors. However, individual sun exposure duration was not assessed. Despite year-round exposure to relatively intense solar radiation in our region, previous studies suggest that sunlight alone may not ensure adequate vitamin D status, as insufficiency and deficiency can also occur in areas with abundant sunlight [103,104,105,106]. Additionally, the universal use of SGLT2 inhibitors among patients with clinical DKD represents a potential confounding factor, as the effect of SGLT2 inhibitors on BP and ND could not be independently assessed due to the absence of variability in treatment exposure across DKD stages.

On the other hand, a major strength of this study is the population uniformity, classified based on DKD stages, with the exclusion of patients undergoing dialysis or in the pre-dialysis stage. Additional strengths include the use of standardized criteria for CAN diagnosis and rigorous validation of ABPM recordings.

Future prospective longitudinal studies are needed to establish the temporal relationship among vitamin D status, impaired nocturnal dipping, CAN, and DKD progression. Larger multicenter studies may help confirm these associations across different stages of kidney and autonomic dysfunction. Moreover, randomized controlled trials investigating whether correction of vitamin D deficiency improves nocturnal blood pressure regulation or autonomic function would be required to determine whether vitamin D has a causal or potentially modifiable role in these abnormalities.

Overall, our results indicate that VD deficiency and the presence of CAN may represent interconnected pathways associated with impaired ND, particularly in advanced stages of DKD. From a clinical perspective, these findings emphasize the significance of a comprehensive cardiovascular assessment in these patients, including the evaluation of CAN and abnormalities in nocturnal dipping patterns, together with biochemical markers such as vitamin D levels, to improve stratification of cardiovascular risk in this group of patients.

## 5. Conclusions

To the best of our knowledge, this study is the first to simultaneously explore the associations among vitamin D concentrations, CAN, and ND across DKD stages in T2DM. Lower serum vitamin D levels and CAN were linked to a higher prevalence of non-dipping in patients with T2DM and advanced DKD. These associations were not observed in early stages, suggesting a stage-dependent interaction between vitamin D status, autonomic dysfunction, and ND. These results highlight ABPM and vitamin D measurement as valuable tools in the assessment of advanced DKD. Although causal inference cannot be established, our results suggest that vitamin D deficiency and autonomic dysfunction may represent interconnected pathways contributing to altered nocturnal blood pressure control and higher cardiovascular risk in this population. Larger longitudinal studies are necessary to confirm our findings.

## Figures and Tables

**Table 1:** Clinical and laboratory parameters of patients with T2DM and early or advanced DKD stage.

Clinical Characteristics	Early DKD Stage	Advanced DKD Stage	*p*
	*n* = 36	*n* = 47	
Age (years)	62.31 ± 6.58	65.53 ± 6.45	0.05
Gender (M/F)	16/20	18/29	0.57
BMI (kg/m^2^)	29.97 ± 4.71	30.01 ± 4.12	0.82
Time since diagnosis of T2DM (years)	10.57 ± 7.10	16.72 ± 8.20	<0.01
Hypertension prevalence (yes%)	28 (77.8)	44 (93.6)	0.03
Dyslipidemia (yes%)	30 (83.30)	44 (93.60)	0.14
Previous cardiovascular event (yes%)	5 (13.90)	5 (10.90)	0.68
History of previous neuropathy (yes%)	18 (50)	44 (93.60)	<0.01
History of retinopathy (yes%)	4 (11.10)	9 (19.10)	0.32
History of smoking (yes%)	11 (30.60)	17 (37.80)	0.50
History of alcoholism (yes%)	12 (33.30)	26 (61.90)	0.01
Autonomic neuropathy (yes total%)	19 (52.80)	32 (72.70)	0.04
Use of ACEi/ARBs (yes total%)	35 (97.20)	47 (100)	0.25
Use of beta-blockers (yes total%)	6 (16.70)	16 (34)	0.08
Use of GLP-1RA (yes total%)	0 (0)	0 (0)	1.00
Use of SGLT2i (yes total%)	0 (0)	47 (100)	<0.01

M: male; F: female; BMI: body-mass index; T2DM: type 2 diabetes mellitus; ACEi: Angiotensin-Converting Enzyme Inhibitors; ARB: Angiotensin II Receptor Blockers; GLP-1RA: Glucagon-Like Peptide-1 Receptor Agonist; SGLT2i: Sodium-Glucose Cotransporter-2 Inhibitors.

**Table 2:** Laboratory parameters of patients with T2DM and early or advanced DKD stage.

Laboratory Features	Early DKD Stage	Advanced DKD Stage	*p*
	*n* = 36	*n* = 47	
25(OH)D (ng/mL)	29.63 ± 7.72	26.86 ± 9.89	<0.05
FPG (mg/dL)	147.39 ± 64.85	162.30 ± 62.10	0.20
Glycated hemoglobin (%)	8.18 ± 1.13	8.21 ± 1.29	0.91
Creatinine (mg/dL)	0.80 ± 0.26	1.46 ± 0.45	<0.01
CKD-EPI (mL/min/1.73 m^2^)	89.22 ± 19.25	51.67 ± 14.76	<0.01
Total cholesterol (mg/dL)	160.03 ± 40.11	182.64 ± 48.31	0.03
HDL (mg/dL)	40.03 ± 10.52	39.91 ± 10.70	0.55
LDL (mg/dL)	85.81 ± 35.92	96.60 ± 41.97	0.24
Triglycerides (mg/dL)	187.92 ± 99.22	227.00 ± 152.03	0.19
Mean albuminuria (mg/g)	105.93 ± 66.03	703.63 ± 834.60	<0.01
Mean albuminuria (log10)	1.95 ± 0.27	2.60 ± 0.50	<0.01

25(OH)D: 25-hydroxyvitamin D; FPG: Fasting Plasma Glucose; CKD-EPI: Chronic Kidney Disease Epidemiology Collaboration; HDL: high-density lipoprotein; LDL: low-density lipoprotein.

**Table 3:** Ambulatory blood pressure monitoring (ABPM) parameters of patients with T2DM and early or advanced DKD stage.

Variables	Early DKD Stage	Advanced DKD Stage	*p*
	*n* = 36	*n* = 47	
24 h SBP (mmHg)	127.58 ± 12.48	138.75 ± 17.45	<0.01
24 h DBP (mmHg)	71.14 ± 7.62	70.45 ± 8.55	0.50
Daytime SBP (mmHg)	129.03 ± 13.00	138.87 ± 16.84	<0.01
Daytime DBP (mmHg)	71.89 ± 8.49	71.26 ± 8.23	0.49
Nighttime SBP (mmHg)	123.06 ± 13.75	137.83 ± 21.00	<0.01
Nighttime DBP (mmHg)	67.31 ± 8.40	67.77 ± 10.63	0.73
Morning SBP (mmHg)	132.84 ± 22.58	145.50 ± 18.21	<0.01
Morning DBP (mmHg)	76.83 ± 14.00	74.29 ± 10.55	0.36
Systolic Dipping (%)	4.51 ± 6.99	0.82 ± 8.38	0.02
Diastolic Dipping (%)	6.38 ± 7.97	5.13 ± 8.12	0.22

SBP: Systolic Blood Pressure; DBP: Diastolic Blood Pressure.

**Table 4:** Comparison between Ambulatory blood pressure monitoring (ABPM) parameters of patients with T2DM advanced DKD stage, according to VD concentrations (Institute of Medicine classification).

Variables	Advanced DKD Stage		*p*
	VD Deficiency	VD Normality	
	<20 ng/mL	≥20 ng/mL	
	*n* = 7	*n* = 40	
Daytime SBP (mmHg)	136.80 ± 17.80	139.20 ± 16.90	0.86
Daytime DBP (mmHg)	69.30 ± 6.80	71.60 ± 8.50	0.49
Nighttime SBP (mmHg)	144.60 ± 22.80	136.60 ± 20.70	0.34
Nighttime DBP (mmHg)	68.80 ± 9.30	67.60 ± 10.90	0.69
Morning SBP (mmHg)	145.90 ± 17.10	145.40 ± 18.60	0.95
Morning DBP (mmHg)	74.90 ± 9.30	74.20 ± 10.90	0.84
Systolic Dipping (%)	−5.60 ± 9.30	1.90 ± 7.80	0.04
Diastolic Dipping (%)	0.75 ± 7.50	5.89 ± 8.10	0.07

T2DM: Type 2 Diabetes Mellitus; DKD: Diabetic Kidney Disease; VD: Vitamin D; SBP: Systolic Blood Pressure; DBP: Diastolic Blood Pressure.

**Table 5:** Ambulatory blood pressure monitoring (ABPM) parameters according to vitamin D status in T2DM patients with advanced-stage DKD (Endocrine Society classification).

Parameters	Advanced DKD Stage			*p*
	VD Deficiency	VD Insufficiency	VD Sufficiency	
	<20 ng/mL	20–29.9 ng/mL	≥30 ng/mL	
	*n* = 7	*n* = 18	*n* = 22	
Daytime SBP (mmHg)	136.80 ± 17.80	138.80 ± 16	139.20 ± 16	0.86
Daytime DBP (mmHg)	69.30 ± 6.80	71.40 ± 7.90	71.70 ± 8.80	0.49
Nighttime SBP (mmHg)	144.60 ± 22.80	139.30 ± 22	134.60 ± 19	0.34
Nighttime DBP (mmHg)	68.80 ± 9.30	68.60 ± 10.90	66.60 ± 10.80	0.69
Morning SBP (mmHg)	145.90 ± 17.10	148.70 ± 19.70	140.70 ± 17.90	0.95
Morning DBP (mmHg)	74.90 ± 9.30	76 ± 10.50	72.10 ± 10.50	0.84
Systolic Dipping (%)	−5.60 ± 9.30	−0.20 ± 7.30	3.30 ± 8	0.04
Diastolic Dipping (%)	0.75 ± 7.50	4.25 ± 7.70	7.23 ± 8.30	0.07

T2DM: Type 2 Diabetes Mellitus; DKD: Diabetic Kidney Disease; VD: Vitamin D; SBP: Systolic Blood Pressure; DBP: Diastolic Blood Pressure.

**Table 6:** Blood pressure parameters across Toronto CAN categories in patients with T2DM and advanced-stage DKD.

Variables	Advanced DKD Stage		*p*
	Absent/Early CAN	Definite/Severe CAN	
	*n* = 28	*n* = 19	
Daytime SBP (mmHg)	139.50 ± 17.50	137.06 ± 15.20	0.65
Daytime DBP (mmHg)	71.25 ± 8.20	71.06 ± 7.80	0.94
Nighttime SBP (mmHg)	135.04 ± 21.60	143.13 ± 19.40	0.22
Nighttime DBP (mmHg)	66.11 ± 11.30	70.75 ± 8.20	0.16
Morning SBP (mmHg)	143.59 ± 19.40	146.23 ± 16.60	0.66
Morning DBP (mmHg)	72.72 ± 9.30	75.79 ± 9.70	0.32
Systolic Dipping (%)	3.34 ± 8	−4.38 ± 7.40	<0.01
Diastolic Dipping (%)	7.58 ± 8.70	0.40 ± 4.80	<0.01

T2DM: Type 2 Diabetes Mellitus; DKD: Diabetic Kidney Disease; CAN: Cardiovascular Autonomic Neuropathy; SBP: Systolic Blood Pressure; DBP: Diastolic Blood Pressure.

## Data Availability

The data presented in this study are available on request from the corresponding author due to being part of an ongoing study.

## Abbreviations

The following abbreviations are used in this manuscript:

ND	Nocturnal Dipping
BP	Blood Pressure
ABPM	Ambulatory Blood Pressure Monitoring
DKD	Diabetic Kidney Disease
CAN	Cardiovascular Autonomic Neuropathy
25(OH)D	25-hydroxyvitamin D
T1DM	Type 1 Diabetes Mellitus
T2DM	Type 2 Diabetes Mellitus
HUJBB	João de Barros Barreto University Hospital
UFPA	Federal University of Pará
UACR	Urine albumin-creatinine ratio
ICF	Informed Consent Form
GFR	Glomerular Filtration Rate
DEQAS	Vitamin D External Quality Assessment Scheme
HbA1c	Hemoglobin A1c
HPLC	High-performance Liquid Chromatography
IOM	Institute of Medicine
CARTs	Cardiovascular autonomic reflex tests
HRV	Heart rate variability
VLF	Very Low Frequency
LF	Low Frequency
HF	High Frequency
TP	Total Power
ACEi	Angiotensin-Converting Enzyme Inhibitors
ARB	Angiotensin II Receptor Blockers
SGLT2	Sodium-Glucose Cotransporter-2
GLP-1	Glucagon-Like Peptide-1
BMI	Body Mass Index
FPG	Fasting Plasma Glucose
CKD-EPI	Chronic Kidney Disease Epidemiology Collaboration
HDL	High-Density Lipoprotein
LDL	Low-Density Lipoprotein
SBP	Systolic Blood Pressure
DBP	Diastolic Blood Pressure
SPSS	Statistical Package for the Social Sciences
ANOVA	One-way Analysis of Variance
RAAS	Renin–angiotensin–aldosterone System
ADA	American Diabetes Association
